# Propranolol induced apoptosis and autophagy *via* the ROS/JNK signaling pathway in Human Ovarian Cancer

**DOI:** 10.7150/jca.46556

**Published:** 2020-08-10

**Authors:** Shujun Zhao, Suzhen Fan, Yanyu Shi, Hongyan Ren, Hanqing Hong, Xiang Gao, Min Zhang, Qiaohong Qin, Hongyu Li

**Affiliations:** 1 Department of Obstetrics and Gynecology, The Third Affiliated Hospital of Zhengzhou University, No.7 Kangfuqian Street, Zhengzhou, 450000, P.R.China.; 2 Zhengzhou Key Laboratory of Gynecological Oncology, 450052 Zhengzhou, China.

**Keywords:** Propranolol, Cell apoptosis, Autophagy, ROS/JNK signaling pathway, Ovarian cancer

## Abstract

Propranolol has a significant anti-cancer effect towards various cancers. Our study aimed at investigating the underlying mechanism of Propranolol's therapeutic effect towards ovarian cancer. Specifically, Propranolol significantly reduced the viability of human ovarian cancer cell lines SKOV-3 and A2780 in a dose- and time-dependent manner. Flow cytometry analysis revealed that Propranolol induced the cell cycle arrest at G2/M phase therefore leading to apoptosis. Moreover, autophagy inhibitor 3-MA markedly enhanced the Propranolol-induced apoptosis. In addition, reactive oxygen species (ROS) increased dramatically after Propranolol treatment and Propranolol activated the phosphorylation of JNK. What is more, p38 inhibitor SB203580 and JNK inhibitor SP600125 attenuated the upregulated expression of LC3-II and cleaved-caspase-3 by the effect of Propranolol. ROS exclusive inhibitor antioxidant N-acetyl cysteine (NAC) weakens the phosphorylation of JNK proteins induced by Propranolol. In summary, these results suggested that Propranolol induced cell apoptosis and protective autophagy through the ROS/JNK signaling pathway in human ovarian cancer cells.

## Introduction

Over the past few decades, ovarian cancer (OC) has become one of the deadliest gynecologic malignancies with the leading cause of cancer-related death among women worldwide, with nearly 140,000 deaths of women occurring every year 1, 2. Cytoreductive surgery and chemotherapeutic drugs are the standard treatment for ovarian cancer. Nowadays, despite significant advances in clinical diagnosis and systemic therapy, the overall 5-year overall survival rate is still less than 30% 3. Therefore, the mechanisms underlying the tumor progression and identification of novel chemotherapy are critical challenges to enhance the therapeutic effect and prolong survival.

Propranolol (PRO) 4 is a well-known non-selective beta-adrenergic receptor (β-AR) antagonist (beta-blocker), which is widely typically prescribed for heart disease, hypertension or angina. In addition, previous preclinical studies 5, 6 have also found that β-AR signaling plays an essential role in cancer progression and the activation of β-AR promotes angiogenesis. A typical β-AR blocker, propranolol has exhibited anticancer effects in various types of solid tumor, including breast cancer 7, hepatocellular Carcinoma 8, melanoma 9 and thyroid cancer 10. Furthermore, the systemic perioperative administration of propranolol was demonstrated effective in reducing tumor burden to women undergoing ovarian cancer surgery 11, suggesting its potential benefits in decreasing tumor growth. Nonetheless, molecular mechanism underlying the anti-cancer effect of Propranolol related autophagy was still remains elusive.

As a particular defective checkpoint in cancer cells, cell cycle deregulation results in genetic modifications, contributing to tumorigenesis 12. The G2/M checkpoint, which functions as cells mitosis stunner, is considered to be a conspicuous target for anticancer drugs. There are many essential proteins involved in G2/M phase transformation, including cyclin B1, cdc2, and p27 13. In addition, Apoptosis and autophagy are known as a major cellular molecular mechanism to regulate cancer progression and maintain the homeostasis. Apoptosis, type-I programmed cell death (PCD), is characterized by specific morphological alter and activated caspases family proteins, which are specific enzymes that rapidly promote signaling cascades and eliminate cellular unserviceable organelles and structures 14. Autophagy is an evolutionarily conserved lysosomal degradation system that excess or aberrant proteins and organelles are delivered into lysosomes to be digested and eventual recycled the resulting macromolecules 15. This process seems to play a pro-survival role in the physiological and pathological processes of cells. However, numerous studies indicated that excessive autophagy resulted in autophagic cell apoptosis, which is known as type II PCD 16, 17. Therefore, autophagy exerts dual effects of pro-survival and pro-apoptosis in cancer cells, which is considered as potent therapeutic targets for the tumor treatment. Thus, the apoptosis and autophagy induced by propranolol need to be further explored.

Next, up-streaming pathways as reactive oxygen species (ROS) and mitogen activated protein kinases (MAPK) 18 are essential for the induction of autophagy and apoptosis. ROS, an active form of diatomic oxygen, has been considered as common by-products of oxidative energy metabolism. Basic levels of ROS may promote cell proliferation and survival, whereas excessive amounts of ROS initiates electron transport chain in mitochondria and activated apoptotic effectors including BAX, Bcl-2, and Cytochrome C, ultimately triggered apoptotic cell death^19^. Besides, Accumulating evidence suggests that the accumulation of intracellular ROS reversibly oxidized autophagy-inducing signaling components to regulate the autophagic activity processes 20. Moreover, ROS has been identified as an inducer or mediator of various intracellular signaling pathways, such as MAPK signaling transduction cascades, which are constituted of c-Jun N-terminal kinase(JNK), p38, and extracellular signal-regulated kinase (ERK)1/2 21, 22. JNK, as stress-activated protein kinase belonging to MAPK family, is a key regulator involved in variety of cellular events, apoptosis and autophagy included. Numerous studies highlight the effect of ROS/JNK on various cancers, such as human osteosarcoma 23, colon cancer 24, lung cancer 25, and human prostate cancer 26. Whether ROS/JNK pathway is associated with the effect of propranolol on human ovarian cancer cells has not been reported.

In this study, we examined whether propranolol induced apoptosis and autophagy through the modulation of the ROS/JNK signaling pathway in human ovarian cancer cells A2780 and SKOV-3. Together, there is an urgent need to elucidate the anti-cancer effect and mechanism of propranolol involved in ovarian cancer cells, which may contribute to develop more effective therapies for human ovarian cancer treatment.

## Materials and Methods

### Reagents and antibodies

Propranolol was obtained from the Chinese materials research center (Beijing, China). Fetal bovine serum (FBS) was got from Gibco, Gaithersburg, MD, USA). N-Acetyl-L-cysteine (NAC), 3-(4, 5-dimethylthiazol-2-yl)-2,5-diphenyl tetrazolium bromide (MTT), Hoechst 33258 staining, and Annexin V-PE Apoptosis Detection Kit were purchased from Beyotime Institute of Biotechnology (Shanghai, China). The enhanced chemiluminescence (ECL) were purchased from Beyotime Institute of Biotechnology (Shanghai, China). JNK inhibitor (SP600125) and 3-MA (3-Methyladenine) were obteined from sigma (St. Louis, MI, USA). Antibodies against Cyclin B1, phospho-cdc2, cdc2, JNK, phosphorylated JNK, and p21 were obtained from Cell Signaling Technology (Beverly, MA, USA). Antibodies against Cdk1, Bcl-2, cleavage caspase-3, cleavage caspase -8, cleavage caspase -9, BAX, LC3-I, LC3- II, Becline-1, p62, GAPDH were purchased from Santa Cruz Biotechnology (Santa Cruz, CA, USA). All other common chemicals and buffers were from Boster (Wuhan, China).

### Cells culture

The human ovarian cancer cell lines A2780 and SKOV-3 were purchased from the Shanghai Institute of Biochemistry and Cell Biology, Chinese Academy of Sciences (Shanghai, China). In this study, the human ovarian cancer cell lines were cultured in a 25 cm^2^ flask with DMEM (Gibco) containing 10% fetal bovine serum (Gibco) at 37 °C/5% CO2 fully humidified atmosphere.

### Cell Viability Assay

The cell viability analysis of A2780 and SKOV-3 cells was performed by MTT assay. Cells were seeded in 96-well plates at a density of 1×10^4^ cells per well overnight. Subsequently, cells were pretreated with Propranolol at different concentrations (0, 80, 100 μM) for 24 h, 48 h, and 72 h, respectively. 20 μl MTT solution was added to each well and incubated for another 4 h at 37 °C. Following 100 μl DMSO dissolved formazan product, the cell viability was measured at 570 nm using a microplate (Molecular Devices, CA, USA). Each experiment was performed for at least three times.

### Cell apoptosis analysis

Cell apoptosis analysis was performed with Annexin V-PE Apoptosis Detection Kit according to the manufacturer's protocols. Briefly, cells were collected and washed twice with cold PBS buffer. Then cells were resuspended in 195μl Annexin V-FITC binding buffer with 5 μl Annexin V-FITC conjugate and 10 μl PI solution for 10~20 min at room temperature. The rate of apoptotic cells was calculated using Cell Quest™ 3.0 software (Becton-Dickinson) and each group was analyzed in triplicate.

### Intracellular ROS detection

The generation of intracellular ROS was assessed using DCFH-DA Cellular ROS Detection Assay Kit (Abcam, USA, Cat. No. ab113851) following the manufacturer's instructions. As intracellular ROS can oxidize non-fluorescent dichlorofluorescein (DCFH) to generate fluorescent compound, dichlorofluorescein (DCF), the test of fluorescence of DCF was able to reflect the level of intracellular ROS. Briefly, following treated with 100 μM Propranolol, cells were incubated with 10 μmol/L non-fluorescent probe DCHF-DA for 20 min at 37 °C in the dark. Then cells were washed twice with ice-cold PBS and examined under flow cytometry (Becton-Dickinson, San Jose, CA, USA).

### Autophagosome detection

To label and monitor the changes of LC3 and autophagy flow, A2780 and SKOV-3 cells were transfected with the mRFP-GFP-LC3 dual fluorescence autophagy indicator system (Hanbio, shanghai, china) following the instructions. Forty-eight hours after transfection, the cells were treated with 100 μM Propranolol for another 24 h. Then the cells were fixed with 4% paraformaldehyde for 15 min at room temperature and washed with ice-cold PBS for three times. The cells with mRFP and GFP dots were quantified under a confocal microscope with 400× magnification. Image J (National Institutes of Health, Bethesda, MD) was used to merge images.

### Western Blot Analysis

The indicated concentrations of Propranolol-treated cells were harvested and lysed in RIPA buffer for 30 min. Cell lysates were centrifuged at 12000g for 10 min at 4 °C and collected the supernatant fraction for immunoblotting. Protein concentrations were determined by Bradford reagent (Bio-Rad, Hercules, CA ). 50 μg of total extracted cell protein/lane were separated on 10% sodium dodecyl sulfate-polyacrylamide gel (SDS-PAGE) and then electrotransferred to polyvinylidene fluoride (PVDF) membranes. After blocked for 3 h with 5% non-fat milk at room temperature, the membranes incubated overnight at 4 °C with the primary antibodies against Cyclin B1, phospho-Cdk1, phospho-Cdc25c, JNK, phosphorylated JNK, p27, Cdk1, Bcl-2, cleavage caspase-3, cleavage caspase -8, cleavage caspase -9, BAX, LC3-I, LC3- II, Becline-1, p62 (all 1:1000 dilution) and GAPDH. Then the membranes were washed with TBST for three times and incubated with horseradish peroxidase-conjugated secondary antibodies (1:5000) for 1 h at room temperature. After washed for three times with TBST, the membranes were immersed in enhanced chemiluminescence (ECL) kit. Immunoreactive proteins were detected by AmershamTM Imager 600 system (GE Healthcare Bio-Sciences, Pittsburgh, PA, USA) and the statistical analysis and densitometry values were estimated by Image J software. GAPDH was used as the loading control.

### Statistical analysis

All data in our work was performed using SPSS software version 16.0 and represented as the mean ± standard deviation (SD). Difference between the treated and the control was determined using the Student's unpaired t-test. P<0.05 was considered as statistically significant.

## Results

### Propranolol suppressed the proliferation of human ovarian cancer cells

**Figure 1:**
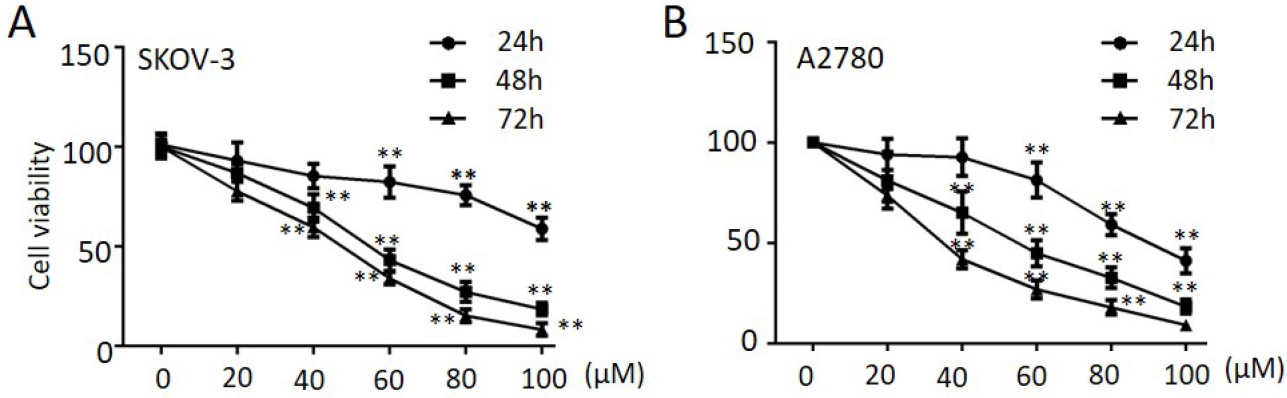
** Propranolol suppressed the proliferation of human ovarian cancer cells.** SKOV-3 (A) and A2780 (B) cells were treated with Propranolol at concentrations of 0, 20, 40, 60, 80 and 100 µM for 24, 48, 72h, respectively. Cell viability was detected by MTT assay. The data are represented as mean ± SD, and obtained from three independent experiments. *P<0.05, **P<0.01, as compared with control.

To explore the anti-tumor effect of propranolol on ovarian cancer cells, SKOV-3 and A2780 were treated with propranolol at various concentrations ranging from 24 h, 48 h and 72 h, respectively. The MTT assay results in Figure 1A & B proved that propranolol dramatically reduced cell viability in a dose-dependent and time-dependent manner. Specifically, the half maximal inhibitory concentration (IC50) values were approximately 190.00, 61.64 and 110.30, 59.66 at 48 h in SKOV-3 cells and in A2780 cells, respectively.

### Propranolol induced cell cycle arrest at G2/M phase in human ovarian cancer cells

**Figure 2:**
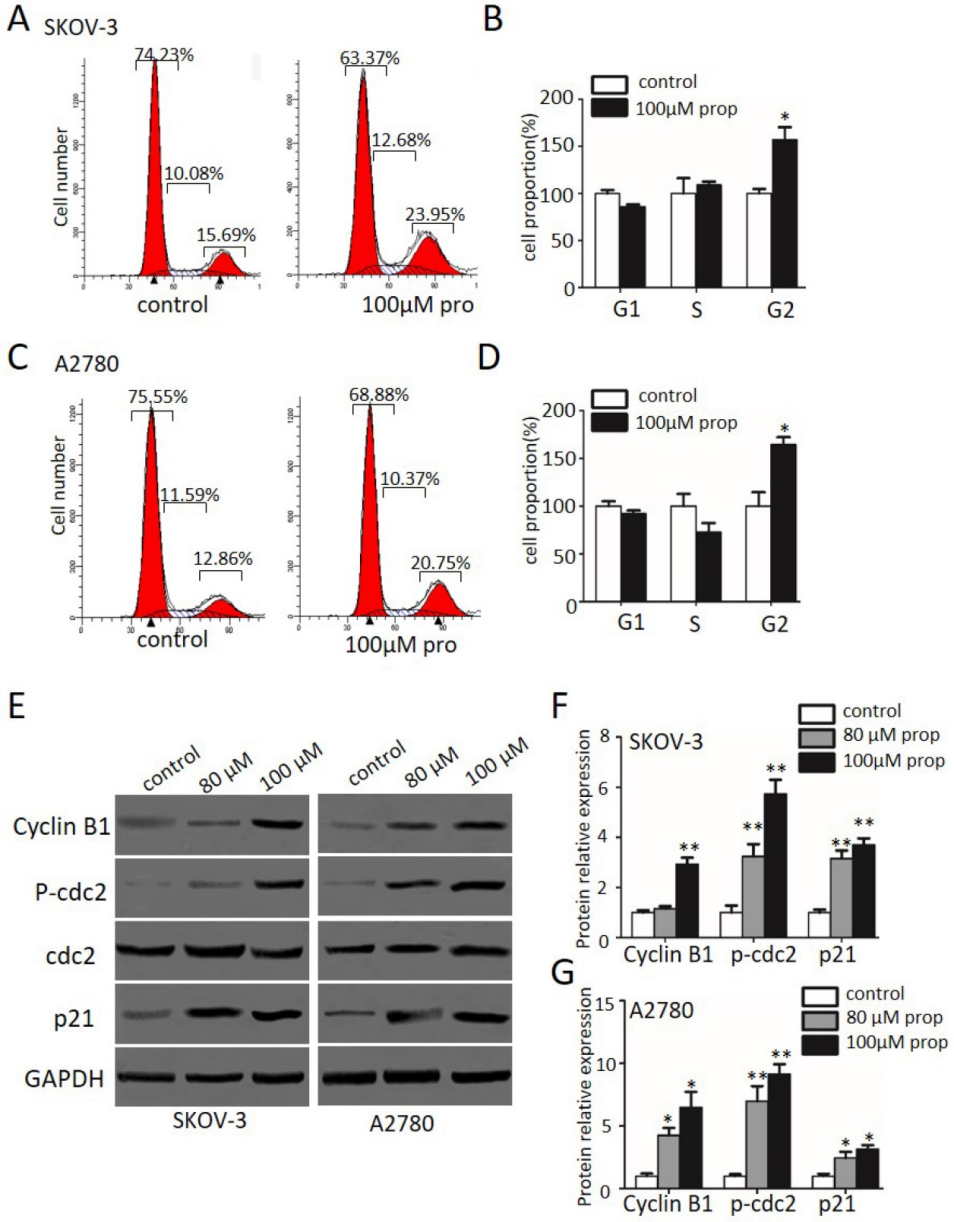
** Propranolol induced cell cycle arrest at G2/M phase in human ovarian cancer cells.** (A and C) Flow cytometry analysis showed the cell cycle distribution when the SKOV-3 and A2780 cells were treated with or without 100 µM Propranolol for 48 hours, respectively. (B and D) Bar graphs show the cell cycle distribution when SKOV-3 and A2780 cells were treated with or without 100 µM Propranolol for 48 hours. Data represent the mean ± SD of three independent experiments. *P<0.05, **P<0.01, as compared with control. (E)The representative blots show the expression levels of Cyclin B1, P-cdc2, cdc2, p21, and GAPDH was used as the internal control. (F)The relative expressions of Cyclin B1, P-cdc2, cdc2, and P21 were quantified by normalizing to GAPDH. All data are represented as mean ± SD, and obtained from three independent experiments. *P< 0.05 vs untreated control group; **P<0.01 vs untreated control group.

In order to determine whether cell cycle arrest is responsible for the cell growth inhibition, we explored the cell cycle distribution detection after exposed to propranolol for 48 h. As shown in Figure 2A-D, cell number in G2/M phase was significantly accumulated in propranolol treated group compared with control group in both SKOV-3 and A2780 cells, implying propranolol induced cell cycle arrest at G2/M phase. Furthermore, the expressions of cell cycle-regulated proteins were investigated by Western Blotting to clarify the underlying mechanisms. The results displayed that propranolol upregulated the expression of Cyclin B1, cdc2, phospho-cdc2, and p27 in SKOV-3 and A2780 cells (Figure 2E-G). All the data above suggested that propranolol triggered G2/M-phase arrest by regulating cell cycle-related proteins in human ovarian cancer cells.

### Propranolol induced apoptosis of human ovarian cancer cells

**Figure 3:**
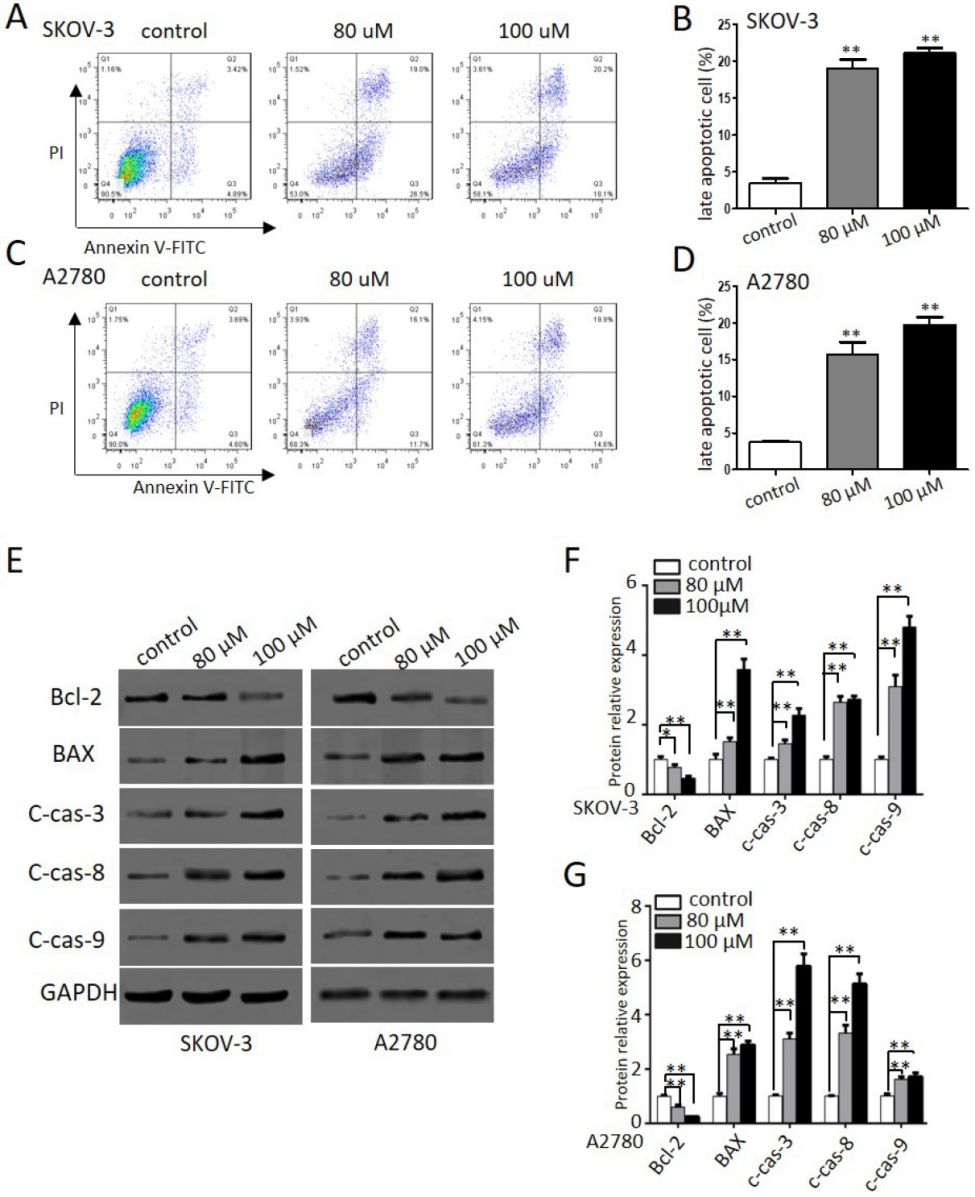
** Propranolol induced apoptosis of human ovarian cancer cells.** (A and C) Flow cytometry showed the different apoptotic stages after the SKOV-3 and A2780 cells treated with 80 µM or 100 µM Propranolol for 48h. (B and D) Bar graphs showed the percentage of late apoptosis when the cells were treated with 80 µM or 100 µM Propranolol for 48 hours. Data are the mean ± SD of three independent experiments. *P<0.05, **P<0.01, as compared with control. (E) Western blot results showed the expression levels of BAX, Bcl-2, cleaved caspase 3, cleaved caspase 8, and cleaved caspase 9 in cells treated with 80 µM or 100 µM Propranolol for 48 hours. (F)The relative expressions of BAX, Bcl-2, cleaved caspase 3, cleaved caspase 8, and cleaved caspase 9 were quantified by normalizing to GAPDH. All data are represented as mean ± SD, and obtained from three independent experiments. **P< 0.01 vs untreated control group.

To further explore the effects of propranolol on apoptosis and cell death in human ovarian cancer cells, we performed flow cytometry and western blotting assay. Figure 3A-D indicated that exposure to propranolol for 48 h led to a dose-dependent increase of apoptotic cells proportion in both SKOV-3 and A2780 cells, whereas the proportion was negligible for control cells. Furthermore, the expression of apoptotic signaling proteins involved in exogenous death receptor pathway and the endogenous mitochondrial pathway mediated by propranolol was performed by western blotting (Figure 3E-G). We found that treating cells with propranolol resulted in an obvious decrease in the Bcl-2 protein expression and the activation of BAX, cleavage caspase-3, -8, and -9, which ultimately triggered the extrinsic and mitochondrial apoptosis pathways. Taken together, these data has elucidated that propranolol provoked apoptotic cell death by activating both extrinsic and mitochondrial pathways.

### Propranolol triggered autophagy inhuman ovarian cancer cells

**Figure 4:**
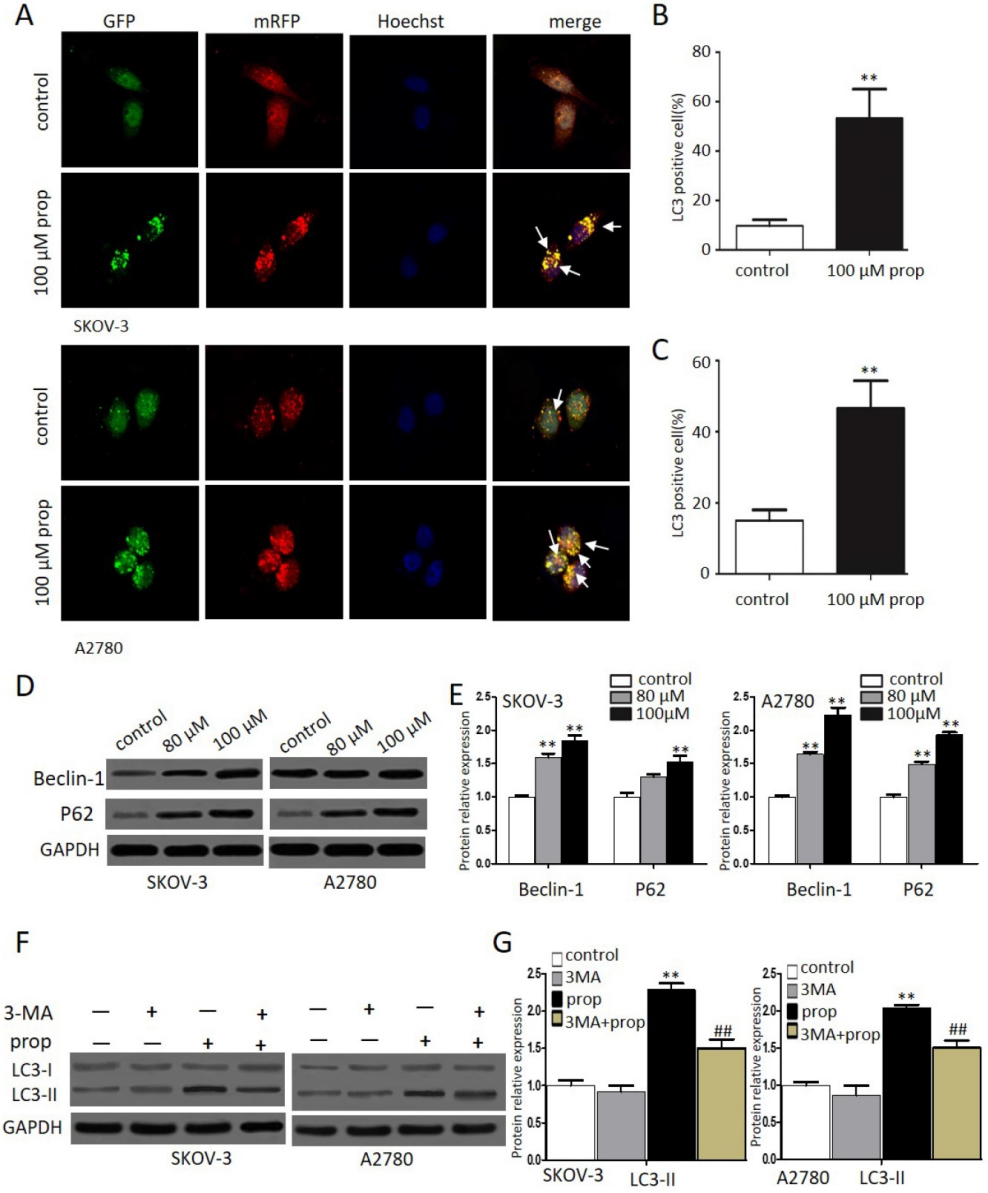
** Propranolol triggered autophagy inhuman ovarian cancer cells.** (A) Representative images of mRFP-GFP-LC3 lentivirus infection monitored the autophagy flux in the SKOV-3 and A2780 cells under confocal microscopy (400×magnification). After merging, white arrows pointed to the LC3 positive cells containing yellow puncta were counted as (B and C) compared with control (**P< 0.01). Then the percentage of LC3 positive cell was calculated relative to all lentivirus infected cells in five different fields under confocal microscopy. Results are counted from three independent experiments and presented as means ± SD. (D) The expression levels of the autophagy-related proteins, including Beclin-1 and P62, were investigated by western blots. (E) The quantitative analysis results of Beclin-1 and P62 were normalized to GAPDH. *P< 0.05, **P< 0.01 vs control group. All data were obtained from three independent experiments. (F) Western blot results showed the expression level of LC3-II in the cells treated with 3MA for 1 hour before incubation with 100 µM Propranolol for 48 hours. (G)The relative expression of LC3-II was quantified by normalizing to GAPDH. All data are represented as mean ± SD, and obtained from three independent experiments. **P< 0.01 vs untreated control group. ##P<0.01 vs Propranolol treatment group.

Autophagy, a form of programmed cell survival, affects tumor initiation and progression. In order to elucidate whether autophagy was involved in propranolol induced cell growth suppression, we investigated autophagic response to propranolol in SKOV-3 and A2780 cells. Autophagy was characterized by an increase in autophagic puncta, which was visualized by LC3II immune fuorescent staining of SKOV-3 cells in culture after 48-hr exposure to propranolol. Autophagy was visualized stably expressing the mRFP-GFP-LC3 (Microtubule associated protein 1 light chain 3) in in the presence of 100 μM propranolol as the arrowhead points in Figure 4A. The statistical results in Figure 4B & C indicated that the number of GFP- and mRFP-LC3 puncta formation was notably increased, suggesting that treatment with propranolol induced autophagy in SKOV-3 and A2780 cells. Subsequently, the expression of several marker protein of autophagy was examined to verify autophagic flu by Western Blot (Figure 4D & E).We found that exposure to propranolol remarkably increased the expression of beclin-1 and p62, which are two key substrates for autophagy, indicating that autophagy was triggered by propranolol treatment.

During autophagy, the conversion of soluble LC3-I to nonsoluble form of LC3-II have been supposed as useful signs of autophagy. Furthermore, to delineate the autophagic flux induced by propranolol, 3‐MA, an inhibitor of autophagy was used to block class III phosphatidylinositol 3-kinases (PI-3Ks) and inhibit the formation of autophagosome in SKOV-3 and A2780 cells. As shown in Figure 4F & G, a marked decrease in the LC3‐II/LC3‐I expression level was observed with 3‐MA pretreatment followed by 100 μM propranolol treatment for 24 h. All of these results indicated that propranolol could induce autophagic flux in SKOV-3 and A2780 cells.

### Autophagy inhibitors promoted propranolol-induced cell apoptosis

**Figure 5:**
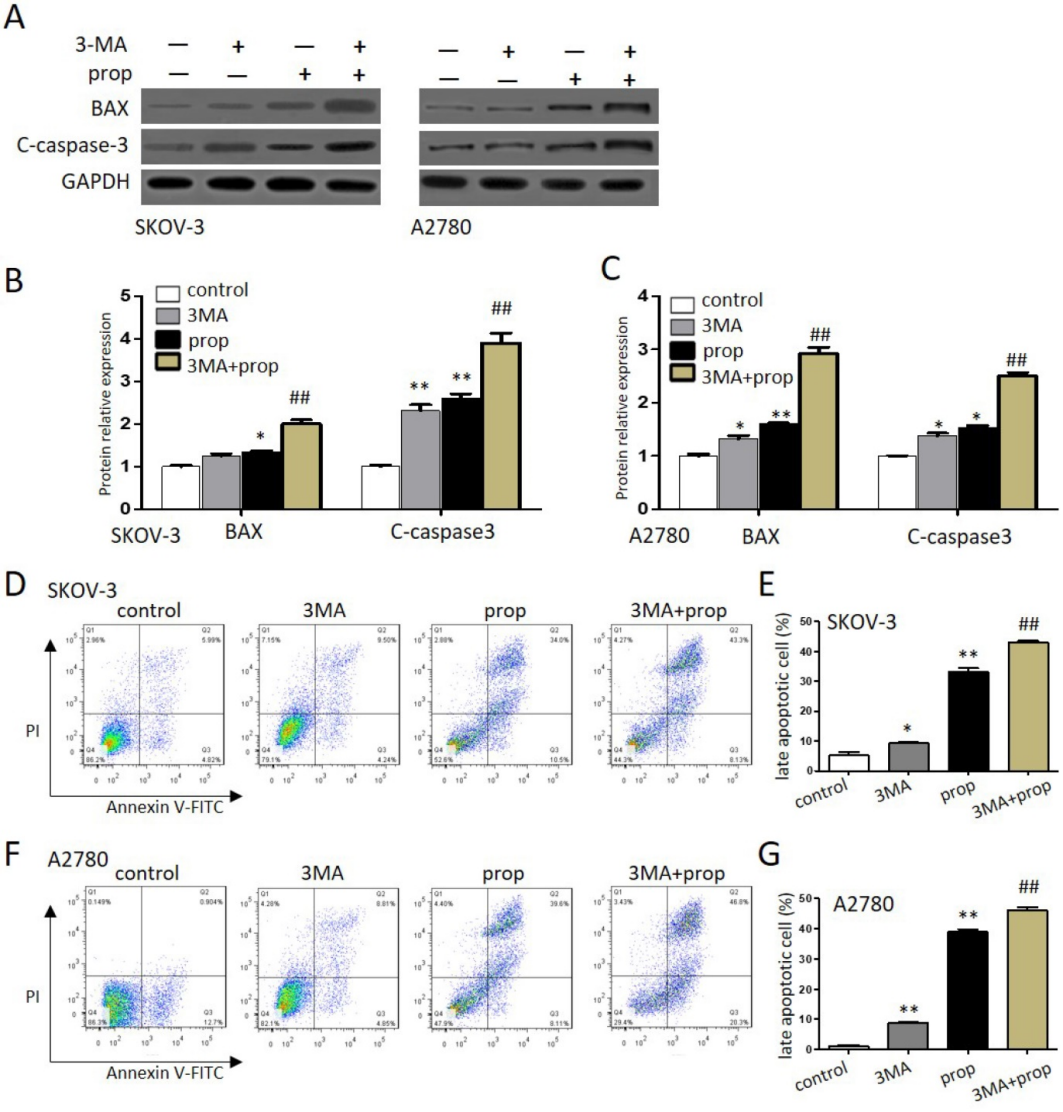
** Autophagy inhibitors promoted propranolol-induced cell apoptosis.** (A) The expressions of BAX and cleaved-caspase 3 were analyzed by western blotting in the cells treated with 3MA for 1 hour before incubation with 100 µM Propranolol for 48 hours in the SKOV-3 and A2780 cells. (B and C) The relative expressions of BAX and cleaved-caspase 3 were quantified by normalizing to GAPDH in the SKOV-3 and A2780 cells, respectively. All data are represented as mean ± SD, and obtained from three independent experiments. **P< 0.01 vs control group. ##P<0.01 vs Propranolol treatment group. (D and F) Flow cytometry showed the different apoptotic stages in the the SKOV-3 and A2780 cells treated with 3MA for 1 hour before incubation with 100 µM Propranolol. Percentage of apoptotic cells were investigated by flow cytometry and quantified as (E and G). **P< 0.01 vs control group. ##P<0.01 vs Propranolol treatment group.

To further investigate either protect cell survival or contribute to cell death that propranolol induced in ovarian cancer cells, autophagy inhibitor 3-MA was employed to explore the results. As shown in Figure 5A-C, the expression of apoptosis‐related proteins BAX and cleaved-caspase‐3 were markedly upregulated by the presence of 100 μM propranolol, and the promoting effects offered by propranolol were further induced by 3-MA in SKOV-3 and A2780 cells, respectively. Moreover, the analysis of flow cytometry (Figure 5D-G) also showed the presence of 3-MA was markedly enhanced the expression of apoptosis proteins (Bax, cleaved-caspase-3) after treated with 100 μM propranolol. These data suggested that cellular reactive autophagy induced by propranolol play a protective effect against apoptosis in ovarian cancer cells.

### ROS generation and JNK activation were triggered by propranolol

**Figure 6:**
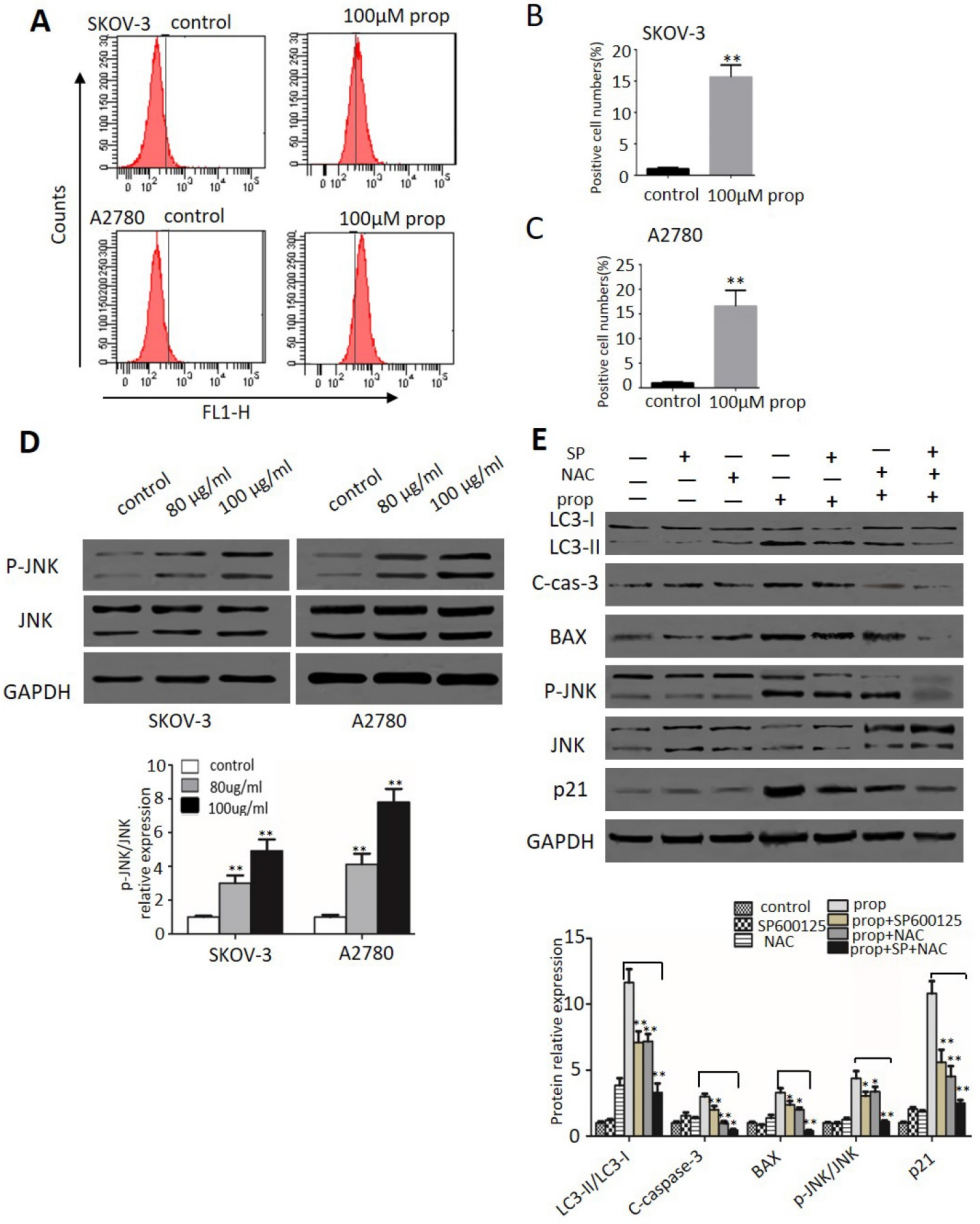
** ROS generation and JNK activation were triggered by propranolol.** (A) SKOV-3 and A2780 cells were treated with 100 µM Propranolol for 48 hours and incubated with DCFH-DA (10 mM) for 20 min at 37°C, then measured by flow cytometer. The percent of positive cell number were calculated as (B) and (C), **P< 0.05 vs untreated control group. (D) The expressions of P-JNK and JNK were analyzed by western blotting in the cells treated with 100 µM Propranolol for 48 hours. (E) The expressions of P-JNK, JNK, BAX, p21, cleaved-caspase 3, and LC3-II were analyzed by western blotting in the cells treated with SP600125 (10 µM) or NAC (5 mM) for 2 hours before incubated with 100 µM Propranolol for 48 hours. *P< 0.05, **P< 0.01.

ROS plays an important role in cancer development and progression, including cell cycle arrest, apoptosis, autophagy as well as sustained JNK 6 cells, 2',7'-dichlorofluorescein-diacetate (DCHF-DA) staining by flow cytometry was applied in our study. As shown in Figure 6A-C, the production of ROS represented by fluorescent signal was dramatically increased when cells exposure to propranolol. Then we investigated the effect of propranolol on JNK pathway. Western blot results in Figure 6D showed that treatment with propranolol notably enhanced the phosphorylation level of JNK in both SKOV-3 and A2780cells. To further confirm the role of ROS/JNK in propranolol-induced cell cycle arrest, apoptosis and autophagy, we preprocessed the cells with the ROS exclusive inhibitor antioxidant N-acetyl cysteine (NAC) and JNK inhibitors SP600125 in the presence/absence of propranolol. From Figure 6E, the Western Blot results showed that both NAC and SP600125 apparently remarkably abolished the increased the protein expression of LC3-II/LC3-I, cleaved caspase-3, BAX, phosphorylated JNK, and p21 by propranolol administration, respectively. Expectedly, NAC concomitant with SP600125 had a harder inhibitory role in propranolol-induced aforementioned protein expression than NAC or SP600125 treatment alone. All these results revealed ROS/JNK pathway activation participated in propranolol-induced cell cycle arrest, apoptosis and autophagy.

## Discussion

The climbing researchers 27, 28 have revealed that propranolol treatment was associated with potent antitumor properties against various human cancers through the inhibition of βreceptors, suggesting propranolol may be functioned as a novel adjuvant therapeutic strategy for tumors. In this study, we discovered propranolol regulated the apoptosis, autophagy through ROS/JNK signal pathway in ovarian cancer cells for the first time.

Firstly, our data demonstrated the significant growth inhibitory effects of propranolol on human ovarian cancer cells via MTT analysis. Anticancer insights were associated with cell cycle arrest, apoptosis and autophagy study have proposed for cancer-cell specific therapeutic strategy. First, flow cytometer assessment was performed to indicate the G2/M phase arrest of SKOV-3 and A2780 cells after propranolol treatment for 48 hours. Some reports 29 have highlighted that cyclin B1 aggregation and Cdc2 phosphorylation were crucial to promote G2/M phase transition. Accordingly, western blot analysis have revealed that propranolol induced G2/M phase arrest via phosphorylated Cdc25C, phosphorylated Cdc2, p27 and downregulation of Cdc2 in both SKOV-3 and A2780 cells.

As a crucial regulator of cancer progression and development 30, apoptosis is characterized by the activation of caspases 31, which are cysteine proteases involved in the initiation and execution proteolytic processing cascade. Accordingly, flow cytometer analysis demonstrated that late apoptotic cells had a dose dependent increase after 100 μM propranolol administration for 48 hours. As initiator caspases, caspase-8 and -9 are the first to be activated in death receptor pathway and mitochondrial apoptosis pathway, respectively. Then this activated the executioner caspase-3 subsequently inducing apoptosis via Bcl-2 and its family members. Western blot assay have demonstrated that propranolol activated pro-apoptotic protein Bax and up regulated the expression of cleaved-PARP, caspase-3, -8, and -9 as well as down regulated Bcl-2 expression. These data indicated that propranolol induced caspase-dependent apoptosis in SKOV-3 and A2780 cells.

In addition, autophagy 32 is also a major intracellular degradation mechanism that regulates cellular homeostasis by maintaining a balance between cell survival and cell death, which is also called “double-edge sword”. On one hand, autophagy 33 decomposed long-lived proteins, damaged organelles and other structures by lysosomal hydrolases to promote survival during various stress conditions. On the other hand, autophagic cell 34 was exerted a similar pro-apoptotic effect when excessive autophagy reaches a certain level. Accumulating findings 35 evidenced the complex paradoxical role of autophagy in antitumor chemotherapy. In our study, we firstly investigated propranolol induced autophagy, as evidenced by the notably accumulation of aggregated autophagosomes following treatment with propranolol. After the process of autophagy initiated, a sequential recruitment of proteins such as beclin-1 and P62 was involved and the conversion of LC3-I to LC3-II was considered as a quantitative indicator of autophagosome formation. The formation of autophagosome membranes has also been identified. Therefore, in the present work, western blot analysis indicated the enhanced the ratio of LC3-II/LC3-I and increased expression of beclin-1 and P62 induced by propranolol correlates with activation of autophagic activity in SKOV-3and A2780 cells. To further ensuring the induction of autophagy flux, SKOV-3 and A2780 cells were pre-incubated with or without autophagic inhibitor 3-MA, which was adopted to block the autophagosome formation at an early stage. Expectedly, pre-incubation with 3-MA significantly reduced the level of LC3-II/LC3-I compared with propranolol treatment alone.

Recently, growing evidence 36 indicate that autophagy is represented as a double‐edged sword for therapeutic purposes in tumor treatment. For example, Jeong-Won Lee et al. revealed that ovarian cancer growth could be abrogated by blocking ADRB-mediated angiogenesis; what is more, the peri-operative use of propranolol could have preventive effects for the surgical stress-induced tumor growth in both HeyA8 and SKOV3ip1 mice models 37. To further clarify the positive or negative effect of propranolol on SKOV-3 and A2780 cells, we found that inhibition of autophagy by 3-MA aggravated propranolol-induced apoptosis. Consistently, the apoptosis related proteins BAX and cleaved-caspase-3 were synchronously induced in the 3-MA+ propranolol group compare compared to the propranolol group alone, revealing that propranolol triggered autophagy acted a protective manner in SKOV-3 and A2780 cells. Therefore, the viewpoint above revealed that antitumor drug combined with 3-MA may be a novel strategy for enhancing antitumor efficacy in chemotherapy of cancers.

The complexity of cell apoptosis and autophagy was associated with ROS generation and concurrently activation of JNK pathways in tumor cells 38. As highly reactive molecules derived from oxidative energy metabolism, ROS levels have crucial role in regulating the fate of cells. Importantly, cellular excessive generation of ROS caused irreversible oxidative damage inducing cell autophagy and apoptosis. In our work, propranolol triggered a significant increase in ROS generation by flow cytometry analysis. Growing evidence 39 has demonstrated that various oxidative stress signals also activated JNK pathway to promote cell apoptosis and autophagy. Western blot results showed that propranolol significantly enhanced the expression of JNK phosphorylation both in SKOV-3 and A2780 cells. Our studies have confirmed that ROS inhibitor NAC and JNK inhibitor SP600125 reversed propranolol-induced increase of LC3-II/LC3-I, cleaved-caspase-3, BAX as well as JNK phosphorylation in SKOV-3 cells. In summary, these data showed that propranolol-induced cell apoptosis and autophagy though the activation of ROS-dependent JNK pathway. Our study provided a new molecular mechanism for the therapeutic effects of propranolol on antitumor therapy.
